# Comparison of Virus Watch COVID-19 Positivity, Incidence, and Hospitalization Rates With Other Surveillance Systems: Surveillance Study

**DOI:** 10.2196/69655

**Published:** 2025-09-29

**Authors:** Wing Lam Erica Fong, Vincent Grigori Nguyen, Sarah Beale, Thomas E Byrne, Cyril Geismar, Ellen Fragaszy, Jana Kovar, Alexei Yavlinsky, Ibrahim Abubakar, Andrew C Hayward, Robert W Aldridge

**Affiliations:** 1 Institute of Health Informatics, University College London, 222 Euston Road, London, NW1 2DA, United Kingdom, 44 02035495969; 2 Institute of Epidemiology and Health Care, University College London, London, United Kingdom; 3 Department of Population, Policy and Practice, UCL Great Ormond Street Institute of Child Health, London, United Kingdom; 4 NIHR Health Protection Research Unit in Modelling and Health Economics, Department of Infectious Disease Epidemiology, School of Public Health, Imperial College London, London, United Kingdom; 5 Department of Infectious Disease Epidemiology, London School of Hygiene & Tropical Medicine, London, United Kingdom; 6 Faculty of Population Health Sciences, University College London, London, United Kingdom; 7 NIHR Health Protection Research Unit in Health Analytics and Modelling, Department of Infectious Disease Epidemiology, School of Public Health, Imperial College London, London, United Kingdom; 8 Institute for Health Metrics and Evaluation, University of Washington, Seattle, United States

**Keywords:** surveillance, COVID-19, SARS-CoV-2, public health monitoring, study comparison

## Abstract

**Background:**

Effective disease surveillance is essential for understanding pathogens’ epidemiology, detecting outbreaks, and enabling timely public health responses. In the United Kingdom, large-scale studies, such as the Office for National Statistics COVID-19 Infection Survey (CIS), have monitored SARS-CoV-2 transmission but required significant resources, making them challenging to sustain when pandemic-specific funding ends and also in resource-limited settings. In contrast, the Virus Watch study, at lower cost, relied on self-reported and linked national testing data as well as symptomatic testing, while Severe Acute Respiratory Infections Watch (SARI) leveraged hospital data for cost-effective surveillance.

**Objective:**

This study aimed to evaluate the effectiveness of Virus Watch as a surveillance system in monitoring COVID-19 positivity, incidence, and hospitalization rates in England and Wales, using data from the CIS and SARI as benchmarks for comparison, while considering the key differences in the study designs, including recruitment strategies, incentives, and testing criteria.

**Methods:**

We used the Virus Watch prospective community cohort study to estimate COVID-19 positivity, incidence, and hospitalization rates in England and Wales from June 2020 to March 2023. Rate estimates were compared with CIS modeled positivity and incidence rates, and with SARI COVID-19 hospitalization rates. Global synchrony between datasets was measured using overall Spearman ⍴ and local synchrony using 9-week rolling Spearman ⍴. For England, comparisons with CIS estimates used Virus Watch rates calculated with and without linked national testing data. Positivity rates were also assessed overall and separately before and after the end of free national testing.

**Results:**

A total of 58,628 participants were recruited into the Virus Watch study, of whom 52,526 (89.6%) were resident in England and 1532 (2.6%) in Wales; region was missing for the remainder. Virus Watch–estimated COVID-19 positivity and incidence rates in England, calculated with and without linked testing data, showed strong global synchrony with CIS estimates (positivity ⍴: 0.91 and 0.90; both *P*<.001 and incidence ⍴: 0.92 and 0.90; both *P*<.001) and strong local synchrony (positivity ⍴: median 0.75, IQR 0.53‐0.85 and median 0.67, IQR 0.47‐0.83, and incidence ⍴: median 0.76, IQR 0.49‐0.88 and median 0.66, IQR 0.45‐0.82), despite having lower absolute values. Global and local synchrony of positivity rates were similar for periods before and after the end of free national testing, although the difference between Virus Watch and CIS estimates was greater post–free testing. COVID-19 hospitalization rates were also lower and less synchronized with SARI estimates. In Wales, Virus Watch estimates exhibited greater variability (positivity ρ: 0.75, *P*<.001; incidence rate ρ: 0.85, *P*<.001) and lower local synchrony (positivity ρ: median 0.61, IQR 0.34‐0.74, and incidence ρ: median 0.52, IQR 0.38‐0.71) compared to England.

**Conclusions:**

Our results highlight the effectiveness of the Virus Watch approach in providing accurate estimates of COVID-19 positivity and incidence rates, even in the absence of national surveillance systems. This low-cost method can be adapted to various settings, particularly low-resource ones, to strengthen public health surveillance and inform timely interventions.

## Introduction

Surveillance systems are essential for epidemic and pandemic response, as they are required for detecting outbreaks and establishing fundamental epidemiological parameters to inform and evaluate effective and efficient public health interventions [1]. Comparing and learning from different surveillance strategies used during the COVID-19 pandemic provides important insights to prepare for future pandemics. In the United Kingdom, large-scale epidemiological studies, such as the Office for National Statistics (ONS) COVID-19 infection survey (CIS) and the Real-Time Assessment of Community Transmission-1 (REACT-1) study, along with sentinel surveillance systems such as Severe Acute Respiratory Infections Watch (SARI), played a crucial role in monitoring SARS-CoV-2 transmission [2,3].

The ONS CIS, considered a “best standard” surveillance study and used as a benchmark in this analysis, involved a substantial investment of £988.5 million (US ~$1.3 billion) since study inception from April 2020 to March 2023 [4]. The study enrolled a representative sample of children and adults (aged 2 years and older) in private residential households (excluding care homes and other communal establishment settings) [2]. They performed weekly nose and throat swabs to test for SARS-CoV-2, irrespective of symptoms. Initially, approximately 150,000 polymerase chain reaction (PCR) tests were conducted monthly in September 2020. By May 2021, the study expanded its testing capacity to analyze an average of 390,300 PCR tests monthly, continuing through March 2022 [2]. To encourage the return of swabs, the ONS CIS offered vouchers as a financial incentive. Up to November 2021, nearly 7 million e-vouchers, which can be spent at a range of retailers online and in-store, worth £211.5 million (US ~$285 million USD) were issued [5]. Despite its valuable insights, ONS CIS is resource-intensive, costly, and requires sufficient testing coverage and capacity, which may be challenging to sustain in many settings, especially where health care resources are limited [6,7].

The Virus Watch study, a community household study conducted in England and Wales, was less resource-intensive, with a total expenditure of £4.89 million (US ~$6.6 million USD). Its design was based on an earlier study tracking influenza transmission, Flu Watch, and it relied on self-reported data and linkage to national COVID-19 testing and hospital admission databases [8,9]. SARS-CoV-2 test results primarily came from free PCR or lateral flow tests (LFTs) made widely available through the national “Test Trace and Isolate Program” launched in May 2020. The program recommended testing for individuals experiencing a high temperature, a new, continuous cough, or a loss or change in their sense of smell or taste or if they have had close contact with someone who has COVID-19, meaning that most testing was symptomatic [10]. No incentives were provided for nasal swab testing. However, reliance on self-reported data and symptomatic testing might limit the accuracy and completeness of the data [9].

In contrast to ONS CIS and Virus Watch, the SARI surveillance system was established in 2020 to monitor hospitalizations and critical care admissions for COVID-19 and other respiratory illnesses (eg, influenza and respiratory syncytial virus [RSV]) across all acute National Health Service (NHS) trusts (hospital trusts that provide secondary health care services) in England [11]. Unlike survey-based systems, SARI is inexpensive and sustainable as it uses existing publicly funded health care infrastructure to estimate the COVID-19 disease burden without extensive community testing [1]. While it only captures data on severe cases of respiratory infections, the data collected is likely to be comprehensive and consistent, offering an overview of epidemiological trends, as around 75% of all acute trusts report hospital and critical care admissions for COVID-19 and other respiratory diseases [12]. However, evaluating only the most severe component of the “clinical iceberg’” of infection may introduce well-recognized bias into key epidemiological parameters, such as potentially overestimating the case fatality rate and underestimating the infection fatality rate, which are critical for informing public health responses [13].

To prepare for future pandemics, it is essential to explore efficient, scalable, and cost-effective methods for collecting and analyzing epidemiological data for acute respiratory disease surveillance that can be implemented in diverse settings. In this analysis, we aimed to evaluate the effectiveness of Virus Watch as a surveillance system in monitoring COVID-19 positivity, incidence, and hospitalization rates in England and Wales. We used data from the CIS and SARI as a benchmark for comparison, while also considering the key differences in the study designs, including recruitment strategies, incentives, and testing criteria.

## Methods 

### Study Design and Participants

Virus Watch was a prospective cohort study that was launched on June 24, 2020, and ran until April 01, 2025. The study recruited 28,527 whole households and 58,628 participants aged 0 to 98 years (with a mean age of 48) up to March 2022 [8]. To participate, households were required to have access to a mobile phone, tablet, or a computer with an internet connection, a valid email address, and at least one household member who could read and respond in English to complete regular surveys. Whole households were required to provide informed consent or, where applicable, assent to participate. Eligible household sizes ranged from one to a maximum of 6 members due to limitations of the REDCap (Research Electronic Data Capture) survey platform (Vanderbilt University) used for data collection. Individuals living in institutional settings, such as care homes and university halls of residence, were not eligible to participate. The Virus Watch cohort was also linked to national COVID-19 testing and vaccination records, and hospital admission and mortality records covering all causes. A detailed description of the study design and methodology can be found in previous publications, with key elements relevant to this analysis outlined here [8,14].

### Identifying SARS-CoV-2 Infections and COVID-19-Related Hospitalizations

Multiple sources were used to identify SARS-CoV-2 infections among Virus Watch participants. These included:

- Linked national testing data: National testing data is obtained from the COVID-19 Second Generation Surveillance system, which contains information on swab tests for SARS-CoV-2, from Pillar 1 (hospital testing) and Pillar 2 (community testing). NHS England linked SARS-CoV-2 test results from both Pillar 1 and 2 to participant data in March 2021. Hospital testing data encompassed results from March 2020 to July 2023, and community testing data from June 2020 to August 2023. The linked data was only available for participants with English postcodes.
- Self-reported test results: Participants reported positive PCR or LFTs in weekly illness surveys. Tests were obtained outside of the study, such as through the UK Test-Trace-Isolate program or privately.
- Virus Watch PCR swab testing: A total of 2 subsets of participants were provided nasopharyngeal swabs for PCR assays for SARS-CoV-2. They self-administered PCR swabs if they experienced symptoms including fever, cough, or loss of taste or smell between October 2020 and May 2021 (n=12,877) and between January and June 2023 (n=2851). All swabs were tested for SARS-CoV-2 RNA via reverse transcriptase–polymerase chain reaction (RT-PCR).

To avoid double-counting tests from the same infection episode, we defined each episode as beginning on the date of the first positive result from any of the data sources. Each episode was assumed to last 90 days from the start date; any subsequent positive tests within the 90-day window were considered part of the same episode. A new episode was only recorded if a positive test occurred more than 90 days after the start of the previous episode. This approach aligns with the UK Health Security Agency definition [15].

We used linked Hospital Episode Statistics Admitted Patient Care data to identify any hospitalizations for or with COVID-19 between March 2020 and March 2024. Hospitalization records provide detailed information on all admissions to NHS hospitals in England, as well as admissions to private or charitable hospitals funded by the NHS. For this analysis, only completed consultations were included. COVID-19-related hospital admissions were identified using the *ICD-10* code U07.1, which indicates confirmed COVID-19. Any admission with U07.1 recorded as a diagnostic code, regardless of whether it was listed as the primary or a secondary diagnosis, was considered a hospitalization for or with COVID-19. Consecutive admissions with a discharge and readmission occurring within a single day were treated as a single admission.

### ONS CIS Modelled Positivity and Incidence Rates

CIS COVID-19 positivity estimates for England were available from April 27, 2020, to January 31, 2023, and incidence rate estimates were available for June 8, 2020, to June 15, 2022. For Wales, positivity estimates were reported from July 27, 2020, to January 31, 2023, and incidence estimates from October 25, 2020 to June 15, 2022 [16].

### SARI Surveillance System Hospitalization Rates

The SARI surveillance system from the UK Health Security Agency provided the overall hospital admission rate of patients with confirmed COVID-19 in England [17]. Hospitalization rates data were available from August 9, 2020, to March 19, 2023.

### Statistical Analysis

#### Virus Watch COVID-19 Positivity, Incidence, and Hospitalization Rates

We calculated 2 weekly positivity and incidence rates: one including linked hospital and community testing data and another based only on self-reported data. Positivity and incidence rates are rates of participants aged 2 years and above, which is in line with ONS CIS study design.

Weekly COVID-19 positivity rates incorporating linked national testing data were calculated as the proportion of participants who tested positive for SARS-CoV-2, identified through either Virus Watch self-reported data or linked national testing data, in a given week, divided by the total number of participants who either completed the weekly Virus Watch survey or had any test result (positive or negative) recorded in linked testing data during that same week. Positivity rates based only on self-reported data were calculated as the proportion of participants who self-reported testing positive in a given week, divided by the total number of participants who responded to the weekly survey during the same week.

The 7-day average incidence rate per 10,000 people, based on self-reported and linked testing data, was calculated by identifying the weekly number of participants who tested positive for SARS-CoV-2 for the first time in ≥90 days. This weekly total, the numerator, was then divided by the weekly number of participants who had either reported no illness in the weekly survey or had only negative SARS-CoV-2 test results recorded in linked testing data in the preceding 90 days. Similarly, the incidence rate based solely on self-reported data were calculated using positive tests reported through weekly surveys. The numerator included participants who self-reported a positive test, having not reported a previous positive in the previous 90 days. The denominator consisted of participants who completed the weekly survey and had either not reported any illness or had not self-reported a positive test in the preceding 90 days. Both incidence rates were divided by 7 to obtain a 7-day average and scaled per 10,000 individuals.

Weekly COVID-19 hospitalization rates per 100,000 people were calculated using the subset of participants with any linked data (hospital records, COVID-19 testing, vaccination, and mortality data). Participants without linked data were excluded from this hospital rate estimation, as their hospitalization status during follow-up could not be determined; their inclusion would risk underestimating rates by inflating the denominator. Hospitalization rates were also restricted to participants in England, as hospital data were not available for Welsh residents. Rates were calculated by identifying the weekly total number of participants hospitalized for or with COVID-19. The denominator included all eligible participants who were alive and under follow-up during each week of the analysis period. Participants entered the denominator from the date of study registration and remained each week until the end of the analysis period or death.

#### ONS CIS Positivity and Incidence Rates

National positivity rates were generated using Bayesian multilevel regression poststratification (MRP) modeling, which were adjusted for potential biases from differences in participation across age, sex, and region. This approach improves the accuracy of positivity estimates by providing a more precise estimate of the population at-risk used as the denominator [18,19]. Nonlinear functions were used to account for time and regional interactions. To estimate COVID-19 incidence rate (per 100,000 people per week), the study first estimated clearance time estimates (the time period someone remains positive). The posterior samples from the MRP models were then combined with the estimated distribution of clearance times, generating incidence estimates of COVID-19 incidence (more details described here [18]).

#### SARI Hospitalization Rates

SARI weekly hospitalization rates are based on the trust catchment population of NHS trusts that actively reported new COVID-19, influenza, or RSV cases for that week [17]. Each week, counts of new COVID-19 admissions to hospitals for all levels of care were summed and converted to rates per 100,000 people based on catchment populations for the trusts for those participating in each reporting week [17].

### Assessing Global and Local Synchrony Between Datasets

We assessed the agreement in trends between Virus Watch estimated and national surveillance data (CIS and SARI estimates) by calculating both global and local synchrony using Spearman ⍴ correlation separately for England and Wales. Spearman ⍴ correlation was used due to the nonparametric nature of the rate estimates. Global synchrony was defined as the overall Spearman ⍴ across the full comparison period, and local synchrony as the median (IQR) of 9-week (roughly 60 d) rolling correlations [20]. Lagged correlations were not tested in this analysis, as the date of SARS-CoV-2 testing was available from both self-reported and linked national testing data. Using the test date as the proxy for infection onset in all cases minimized the potential for temporal misalignment between datasets.

For England, we compared Virus Watch positivity estimates calculated with linked national testing data to CIS positivity estimates, and we also compared Virus Watch positivity estimates calculated from only self-reported testing data to CIS positivity estimates. These comparisons with CIS estimates were repeated separately for the periods before the end of free national testing (June 22, 2020‐March 31, 2022) and after the end of free national testing (April 1, 2022‐January 31, 2023), for both linked and unlinked Virus Watch positivity estimates. In addition, we compared Virus Watch incidence rates calculated with linked national testing data to CIS incidence estimates, and Virus Watch incidence rates based on self-reported testing data only to CIS incidence estimates. We did not conduct comparisons with CIS estimates pre- and post-testing withdrawal for incidence rates, as the post-testing period was too short (April 1, 2022‐June 20, 2022) to calculate meaningful rolling correlations. Finally, we compared Virus Watch–estimated COVID-19 hospitalization rates with SARI estimates between August 9, 2020, and March 19, 2023.

For Wales, only self-reported testing data were available; therefore, we compared self-reported positivity and incidence rates with CIS estimates. We repeated positivity comparisons with CIS estimates before (June 22, 2020‐March 31, 2022) and after the end of free national testing (April 1, 2022‐January 31, 2023). No pre- and post-testing withdrawal comparisons with CIS estimates were conducted. Furthermore, linked hospitalization data were not available for Wales; therefore, no comparisons were made with SARI.

Weekly survey response data were extracted from REDCap, linked, and analyzed in R (version 4.4.1; R Foundation for Statistical Computing) and RStudio (version 2025.05.1+513; Posit).

### Ethical Considerations

Virus Watch was approved by the Hampstead NHS Health Research Authority Ethics Committee (20/HRA/2320) and conformed to the ethical standards set out in the Declaration of Helsinki. Participants provided informed consent and, where relevant, for children that they were responsible for, for all aspects of the study. The survey dataset was deidentified by removing participants’ names, dates of birth, and residential addresses. These were stored in another dataset with matched study IDs to preserve anonymity. All datasets were stored in a secure remote server and only accessed by designated research staff and investigators.

## Results

### Overview

The Virus Watch study recruited 58,628 participants between June 2020 and February 2023, primarily residents of England (52,529/58,628, 89.6%) with a smaller proportion (1521/58,628, 2.6%) from Wales. Compared to the general population, the study cohort was older, with a higher representation of individuals aged 45‐64 years and 65 years and older. While the cohort was generally balanced in terms of sex, there were notable disparities in regional and ethnic representation. Certain regions, such as the North West, South West, West Midlands, and Yorkshire and the Humber, were underrepresented, while others, like the East of England and South East, were overrepresented. Ethnic minority groups, including Black and other Asian populations, were also underrepresented in the study (see Table 1).

**Table 1.** Demographic characteristics of Virus Watch study participants at baseline recruitment compared to the Office for National Statistics (ONS) COVID-19 Infection Survey (CIS) and 2021 census.

Characteristic	Virus Watch participants (n=58,628)	ONS CIS (n=535,121) [2]	Percentage of England population (ONS census), % [21,22]
Age group (y), n (%)			
2‐11	4617 (7.9)	33,507 (6.3)	13
12‐16	2623 (4.5)	29,147 (5.4)	6
17‐24	3000 (5.1)	35,403 (6.6)	9
25‐34	4973 (8.5)	52,359 (9.8)	14
35‐49	10,406 (17.7)	103,068 (19.3)	20
50‐69	22,789 (38.9)	176,814 (33.0)	25
70+	9589 (16.4)	104,823 (19.6)	14
Sex (including derived)**^a^**, n (%)			
Male	26,274 (44.8)	252,047 (47.1)	49
Female	31,533 (53.8)	283,074 (52.9)	51
Other or Missing or Prefer not to say	821 (1.4)	—^b^	—
Ethnicity, n (%)			
White	43,968 (75.0)	493,026 (92.1)	86
Asian/ Asian British	3155 (5.4)	22,128 (4.1)	7
Black, African, Caribbean, or Black British	493 (0.8)	5476 (1.0)	3
Mixed or multiple ethnic groups	998 (1.7)	9598 (1.8)	2
Other ethnic groups	288 (0.5)	4804 (0.9)	2
Missing	9726 (16.6)	89 (0.01)	—
Region, n (%)			
North East	2528 (4.3)	—	5
North West	5572 (9.5)	—	12
Yorkshire and The Humber	3035 (5.2)	—	9
East Midlands	4945 (8.4)	—	8
West Midlands	3020 (5.2)	—	10
East of England	10,545 (18.0)	—	11
London	9083 (15.5)	—	15
South East	9845 (16.8)	—	15
South West	3956 (6.7)	—	10
Wales	1532 (2.6)	—	5
Missing	4567 (7.8)	—	—

a Sex at birth was self-reported. If missing, sex was supplemented via data linkage or derived via name-sex matching based on US names from 1930‐2015 [23].

b Not available.

The study identified a total of 30,031 COVID-19 cases in England and 570 in Wales between June 2020 and February 2023. These cases were identified through self-reported positive PCR or LFT results (n=28,463), positive results from Virus Watch swabs (n=92), and data linkage (n=29,609). It is important to note that these sources were not mutually exclusive, meaning some cases may have been identified through multiple sources. Among the 35,551 participants with linked data, 642 COVID-19-related hospitalizations were identified.

### Assessing Global and Local Synchrony Between Datasets

**Figure 1.**
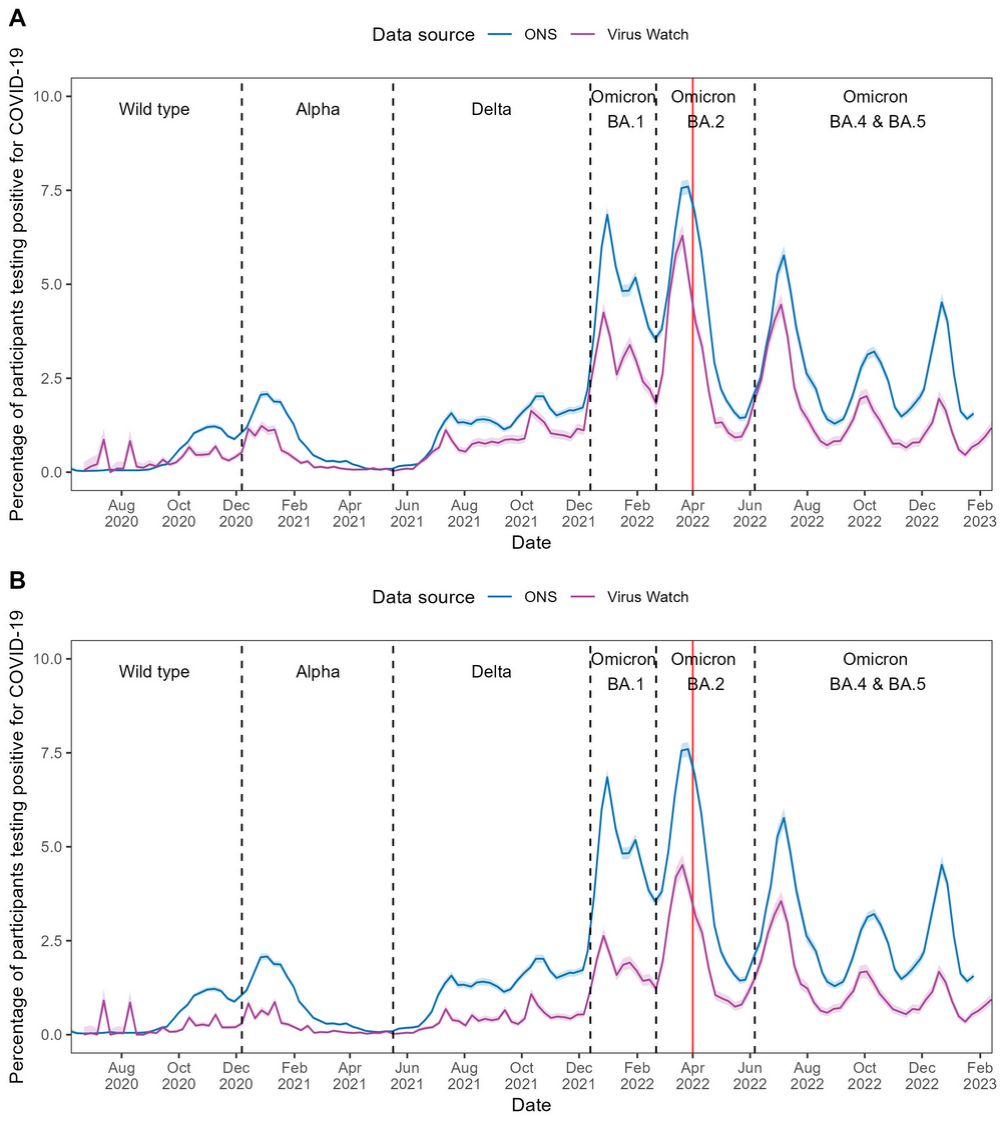
COVID-19 positivity rates in England estimated using Office for National Statistics (ONS) COVID-19 Infection Survey (CIS) and Virus Watch data from June 2020 to February 2023, with the dominant variant of concern for each period indicated. Panel A presents Virus Watch estimates calculated with linked national testing data, while Panel B presents Virus Watch estimates calculated without linked national testing data. Shaded areas represent 95% CIs for the positivity rate estimates, and the red line denotes the end of free national COVID-19 testing.

CIS and Virus Watch–estimated positivity rates calculated with linked national testing data exhibited similar trends across all waves of infection, although Virus Watch consistently reported lower peaks, particularly during the Omicron BA.1 wave (see Figure 1A). Overall, Spearman ⍴ correlation demonstrated high global synchrony between Virus Watch and CIS estimates, with ⍴=0.92 (*P*<.001). The 9-week rolling correlations also demonstrated strong local synchrony, with a median ⍴ of 0.75 (IQR 0.53‐0.85; see Table 2 and Figure S1 in Multimedia Appendix 1). Assessing synchrony separately before and after the end of free national testing, before the end of free national testing, strong global synchrony (⍴=0.89; *P*<.001) was observed, alongside a 9-week rolling correlation median ⍴ of 0.72 (IQR 0.47‐0.85; see Table 2 and Figure S1 in Multimedia Appendix 1). Following the end of free testing, the global synchrony decreased (⍴=0.74; *P*<.001), while local synchrony remained similar to before the end of free national testing (median ⍴=0.76, IQR 0.58‐0.88).

**Table 2.** Overall and 9-week rolling correlation between Virus Watch and Office for National Statistics (ONS), COVID-19 Infection Survey (CIS), and Severe Acute Respiratory Infections Watch (SARI) estimates. The median (IQR) of correlation coefficients was reported for the 9-week rolling correlation. The time period indicates when each correlation was measured.

Region and rate estimates	Time period	Overall		9-week rolling
		⍴	*P* value	median ⍴ (IQR)
England				
Positivity rates with linked data				
Overall	June 22, 2020-January 31, 2023	0.91	<.001	0.75 (0.53‐0.85)
Before end of free national testing	June 22, 2020-March 31, 2022	0.89	<.001	0.72 (0.47‐0.85)
After end of free national testing	April 01, 2022-January 31, 2023	0.74	<.001	0.76 (0.58‐0.88)
Positivity rates without linked data				
Overall	June 22, 2020-January 31, 2022	0.90	<.001	0.67 (0.47‐0.83)
Before end of free national testing	June 22, 2020-March 31, 2022	0.87	<.001	0.57 (0.40‐0.76)
After end of free national testing	April 01, 2022-January 31, 2023	0.72	<.001	0.72 (0.53‐0.83)
Incidence rates with linked data	June 22, 2020-June 20, 2022	0.91	<.001	0.77 (0.53‐0.85)
Incidence rates without linked data	June 22, 2020-June 20, 2022	0.90	<.001	0.66 (0.45‐0.82)
Hospitalization rates	August 09, 2020-March 19, 2023	0.74	<.001	0.49 (0.15‐0.70)
Wales				
Positivity rates				
Overall	July 27, 2020-March 31, 2022	0.77	<.001	0.63 (0.40‐0.76)
Before end of free national testing	July 27, 2020-March 31, 2022	0.67	<.001	0.55 (0.26‐0.70)
After end of free national testing	April 01, 2022-January 31, 2022	0.63	<.001	0.68 (0.51‐0.82)
Incidence rates	October 26, 2020-June 20, 2022	0.86	<.001	0.52 (0.38‐0.71)

Without linked testing data, Virus Watch–estimated positivity rates were lower than estimates calculated with linked data. Compared to CIS estimates, overall trends remained broadly similar but showed less alignment during the Wild type, Alpha, and Delta waves. However, overall, Virus Watch positivity estimates were consistently lower than those from CIS (see Figure 1B). Across the full study period, Virus Watch estimates without linked testing data maintained strong global synchrony with CIS estimates (positivity ⍴=0.90; *P*<.001). The 9-week rolling correlation was comparatively weaker (median ⍴=0.67, IQR 0.47‐0.83) than for comparisons using rates calculated with linked data; yet, trends over time remained similar (see Figure 1B, Table 2, and Figure S1 in Multimedia Appendix 1). Global synchrony was stronger before the end of free national testing than after, while local synchrony was higher after free national testing than before (see Table 2).

**Figure 2.**
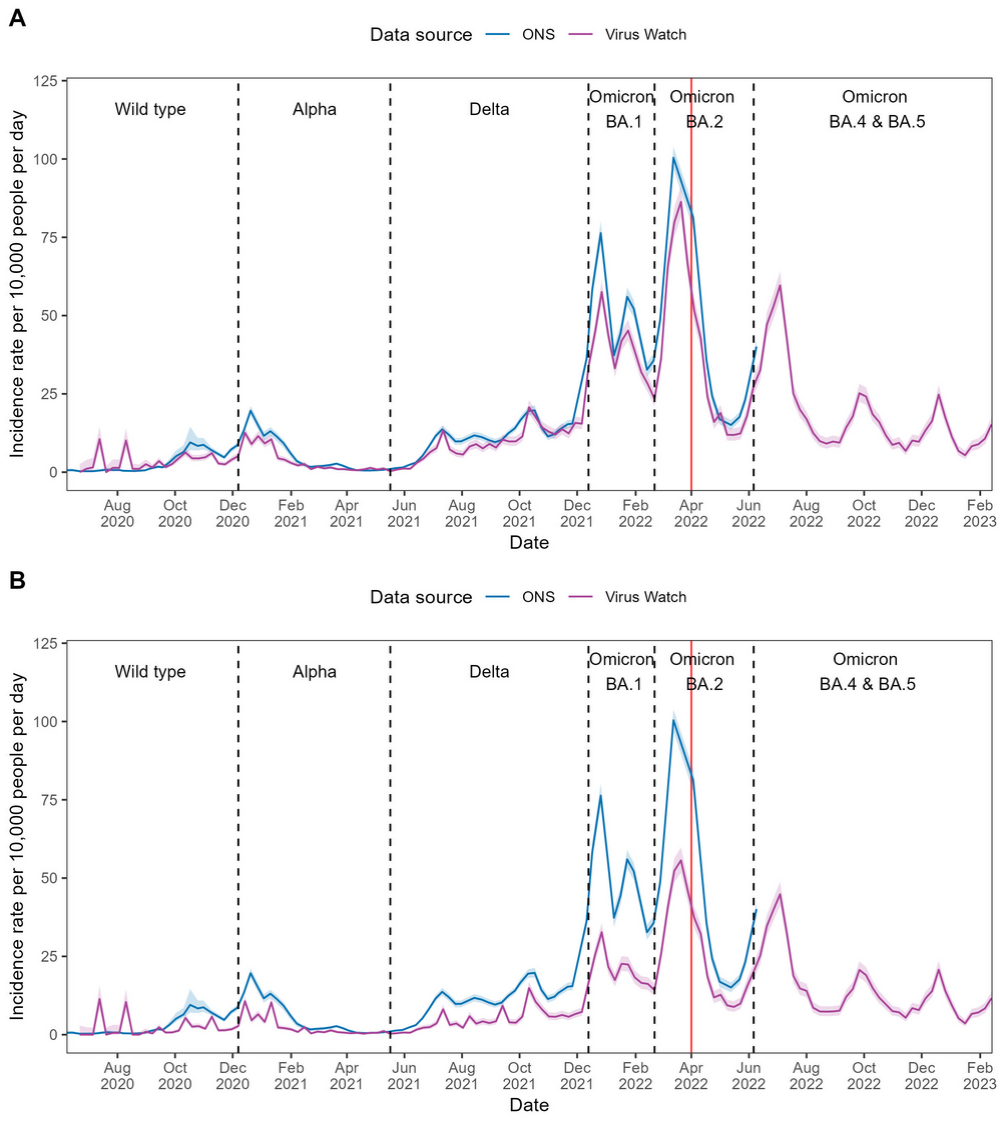
COVID-19 incidence rates in England estimated using Office for National Statistics (ONS) COVID-19 Infection Survey (CIS) and Virus Watch data from June 2020 to February 2023, with the dominant variant of concern for each period indicated. Panel A presents Virus Watch estimates calculated with linked national testing data, while Panel B presents Virus Watch estimates calculated without linked national testing data. Shaded areas represent 95% CIs for the incidence rate estimates, and the red line denotes the end of free national COVID-19 testing.

In England, Virus Watch–estimated incidence rates calculated with linked national testing data demonstrated strong concordance with CIS estimates across all waves of infection (see Figure 2A). While the overall magnitude of both rates was generally comparable, Virus Watch–estimated peaks during the Omicron BA.1 and BA.2 waves were slightly lower than in the CIS estimates. Spearman ⍴ correlation demonstrated high global synchrony between Virus Watch and CIS estimates (⍴=0.91; *P*<.001). The 9-week rolling correlation also indicated strong local synchrony, as seen by a high median ⍴ (median 0.76, IQR 0.49‐0.88; see Table 2 and Figure S1 in Multimedia Appendix 1). Without linked national testing data, the Virus Watch-estimated incidence rates were consistently lower than those of CIS (see Figure 2B). However, high global synchrony (⍴=0.90; *P*<.001) was maintained, demonstrating similar temporal trends. The strength of local synchrony was reduced compared with estimates including linked data, but still reflected moderate agreement (median ⍴=0.66, IQR 0.45‐0.82) (see Table 2 and Figure S1 in Multimedia Appendix 1).

**Figure 3.**
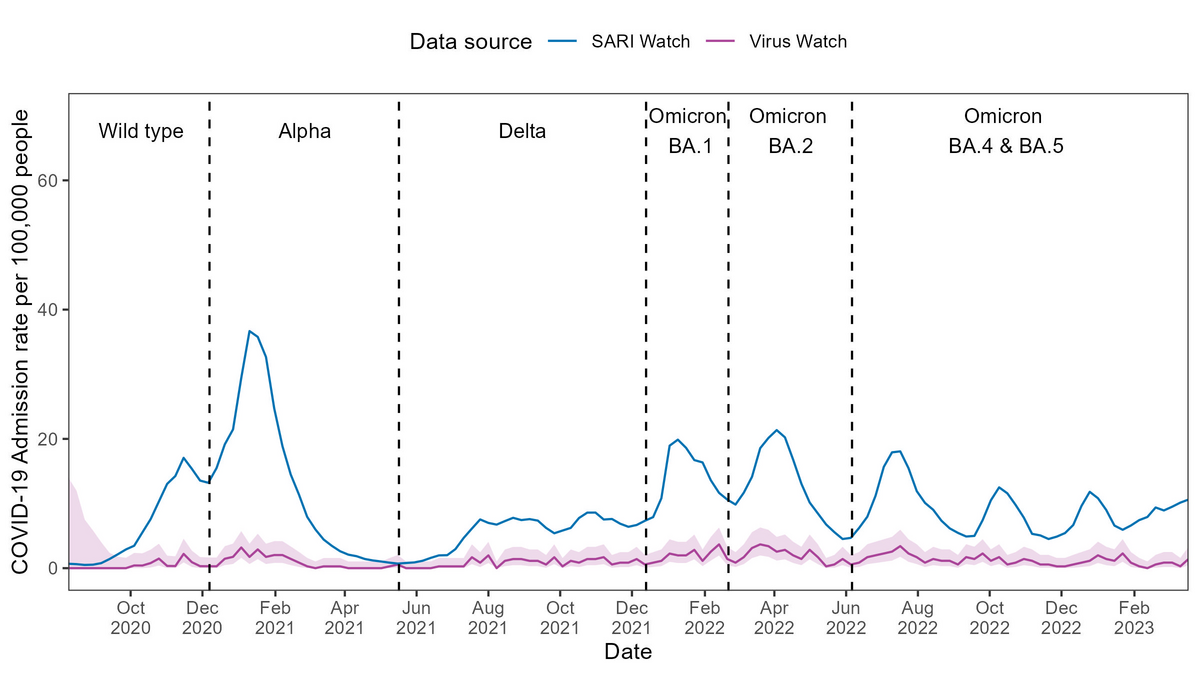
COVID-19 hospitalization rates in England estimated using Severe Acute Respiratory Infections Watch (SARI) and Virus Watch data from June 2020 to March 2023, with the dominant variant of concern for each period indicated. Shaded areas represent 95% CIs for the hospitalization rate estimates.

The Virus Watch–estimated hospitalization rates with COVID-19 were significantly lower than SARI estimates and did not follow the same trend, failing to capture any peaks of admission observed in the SARI data across all waves of infection (see Figure 3). While Spearman ⍴ correlation showed an overall positive correlation with SARI estimates (⍴=0.74; *P*<.001), the 9-week rolling correlation showed weaker correlation and greater variability (median ⍴=0.49, IQR 0.15‐0.70; see Table 2 and Figure S2 in Multimedia Appendix 1).

**Figure 4.**
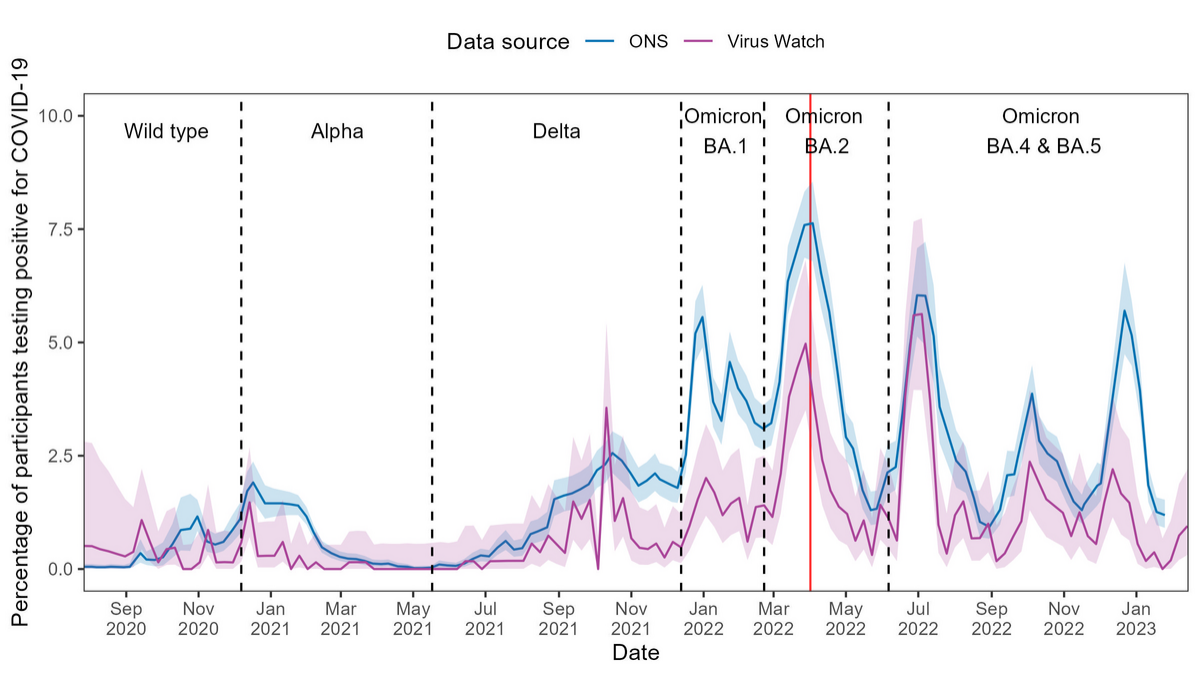
COVID-19 positivity rates in Wales estimated using Office for National Statistics (ONS) COVID-19 Infection Survey (CIS) and Virus Watch data from June 2020 to February 2023, with the dominant variant of concern for each period indicated. Shaded areas represent 95% CIs for the positivity rate estimates, and the red line denotes the end of free national COVID-19 testing.

**Figure 5.**
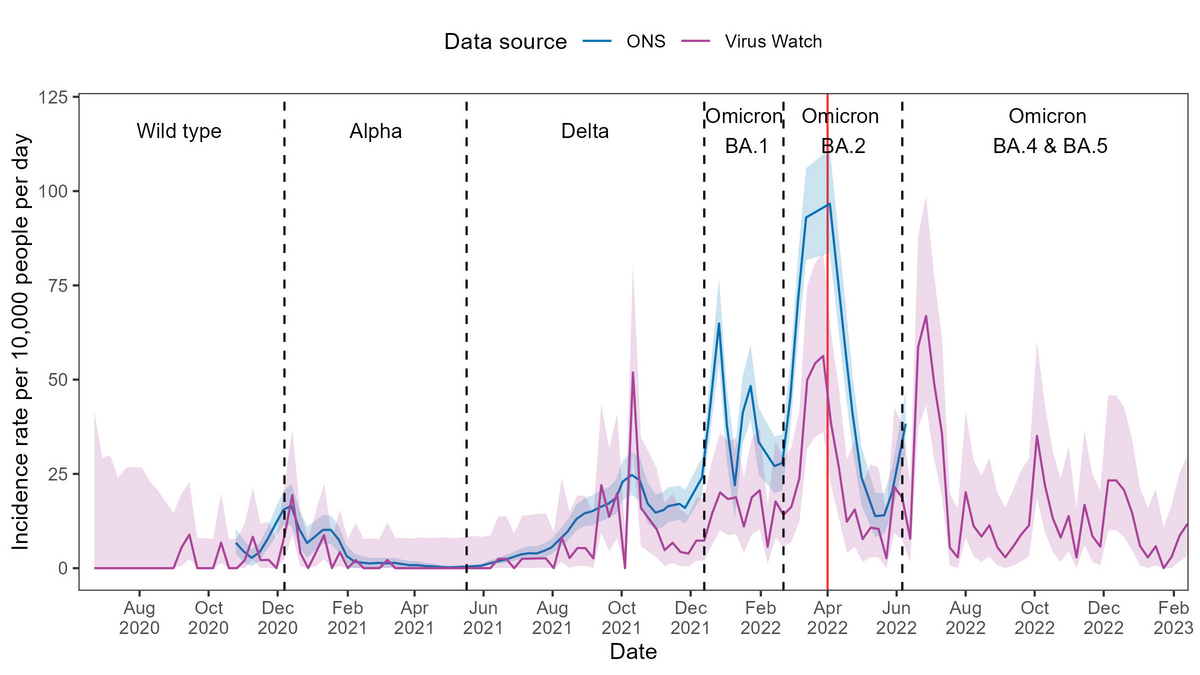
COVID-19 incidence rates in Wales estimated using Office for National Statistics (ONS) COVID-19 Infection Survey (CIS) and Virus Watch data from June 2020 to February 2023, with the dominant variant of concern for each period indicated. Shaded areas represent 95% CIs for the incidence rate estimates, and the red line denotes the end of free national COVID-19 testing.

In Wales, Virus Watch-estimated positivity (see Figure 4) and incidence (see Figure 5) rates exhibited greater variability and wider confidence intervals compared to CIS estimates. Both estimates demonstrated broadly similar temporal trends to CIS estimates, with peaks corresponding to major waves of infection. However, Virus Watch estimates were consistently lower than those from CIS. For positivity rates, the difference between the two estimates appeared largest during the Omicron BA.1 wave, which narrowed during Omicron BA.2 and later waves. The overall trend of Virus Watch positivity rates was strongly correlated with CIS estimates (⍴=0.77; *P*<.001), while a median ⍴ of 0.63 (IQR 0.40‐0.76) suggested moderate local synchrony. Global synchrony was stronger in the period before the end of free national testing, whereas local synchrony was higher after free testing ended (see Table 2). Furthermore, the overall Spearman ⍴ for incidence rate comparison indicated high global synchrony between the 2 estimates (⍴= 0.86; *P*<.001). Local synchrony (median ⍴=0.52, IQR 0.38‐0.71) indicated a moderate correlation, lower than that observed for incidence rate comparisons restricted to England (see Table 2).

## Discussion

### Principal Findings

Using Virus Watch data, we estimated COVID-19 positivity, incidence, and hospitalization rates in England and Wales, comparing our findings with CIS and SARI estimates. In England, we observed a strong overall and 9-week rolling correlation between Virus Watch and CIS positivity and incidence rates, particularly when using Virus Watch estimates with linked national testing data. Correlations were weaker for Wales, likely due to fluctuations in the Virus Watch rates driven by the small, nonrepresentative Welsh cohort and the lack of linked national testing data for this region. For both countries, global synchrony of positivity rates was stronger before the free testing ended, while local synchrony was stronger after, possibly reflecting greater disagreement in early pandemic trends when testing access and behaviors were less established. Correlations between Virus Watch and SARI hospitalization rates were also positive but weaker than those observed with CIS positivity and incidence estimates.

The feasibility and effectiveness of internet-based, population-based surveillance studies were also demonstrated early in the pandemic through the COVID-19 Symptom Study, which recruited over 2.8 million users and received more than 120 million daily reports of symptoms or swab test results on their mobile app [20]. Although the study did not link to national testing data, it was still able to estimate COVID-19 prevalence, incidence, and effective reproduction number that were in good agreement with estimates from CIS. These findings reinforce the potential of participatory surveillance to track population-level infection patterns.

### Key Differences in Study Designs and Their Implications

ONS CIS, SARI, and Virus Watch differ significantly in study designs, resulting in Virus Watch reporting lower COVID-19 positivity, incidence, and hospitalization rates than CIS and SARI. CIS, with its large, population-based sample and regular and incentivized PCR testing, captured both symptomatic and asymptomatic cases [2]. Virus Watch, by contrast, primarily captured symptomatic cases through self-testing, with incentives provided only during a specific recruitment phase [8]. Without continuous incentives for engagement and following the end of free national testing, Virus Watch yielded lower estimates of COVID-19 positivity and incidence. For SARI, data reporting is mandatory from NHS trusts; however, between 2023 and 2024, about 75% of trusts contributed data [12]. Although trust coverage is incomplete, SARI is still able to provide indicative estimates of hospitalization rates in England [12]. Virus Watch hospitalization data was obtained through linkage to national hospital surveillance data, which reduces the risk of underreporting compared to self-report. However, since the cohort is not fully representative of the general population, particularly underrepresenting individuals who are very old or clinically extremely vulnerable, Virus Watch–estimated hospitalization rates are lower than those reported by SARI.

Surveillance systems are essential for timely outbreak detection, identifying at-risk populations, and evaluating population-level public health interventions. In addition, factors such as timeliness and cost-effectiveness should be considered for their implementation [20]. While the CIS can be considered the “best standard” surveillance system, its limitations, including timeliness and cost, can restrict its implementation in any setting once pandemic-specific funding ends and may pose even greater challenges in resource-limited settings [20]. Passive surveillance systems, such as SARI, are relatively inexpensive and easy to implement as they use real-time data from existing publicly funded hospital-based systems [1]. However, SARI estimates, similar to Virus Watch estimates, are contingent on national testing guidelines and practices, which may impact its sensitivity [12]. Furthermore, while SARI remains a reliable surveillance system for monitoring hospitalizations due to respiratory infections, its usability for tracking disease spread within the community using hospitalization rates may be affected, particularly as the clinical pyramid of infection evolves. For example, vaccination rollouts and the emergence of less virulent variants can reduce disease severity, resulting in fewer admissions despite ongoing community transmission [20]. In addition, the determinants of exposure can differ substantially from those of severe disease requiring hospitalization. Using only hospitalization data to monitor disease spread without understanding patterns of community transmission creates an information bottleneck in epidemic and pandemic contexts. It can also overestimate virulence, distorting key epidemiological metrics and public health strategies [13]. Alternatively, surveillance using national testing data has been shown to be a reliable indicator of epidemic progress and offers timely and extensive coverage. However, it may lack the information necessary to identify at-risk populations and risk factors for infection [24]. The reliance on a comprehensive national testing infrastructure also limits the viability of this approach.

Given the limitations of individual surveillance systems, a complementary approach, combining multiple methods, is preferable. Virus Watch used a multifaceted approach that integrated community-based testing, internet-based weekly surveys, and linkage to national datasets to estimate COVID-19 positivity, incidence, and hospitalization rates. Data linkage reduced the cost of the study as well as the risk of underreporting associated with self-testing, enhancing the study results’ reliability. However, while Virus Watch hospitalization estimates are less suitable for tracking absolute rates, they can provide some indication of trends over time and remain valuable for identifying relative differences across demographic groups.

The collection of sociodemographic and clinical data of participants also allowed for the analysis of clinical risk factors and symptom profiles across different SARS-CoV-2 variants [25-31]. The Virus Watch approach can act as a low-cost supplementary surveillance method during periods of national testing. Without a national testing program, Virus Watch’s symptomatic self-swabbing strategy provides reliable estimates for community positivity and incidence rates at significantly lower costs compared to larger studies that involve swabbing regardless of symptoms. Our analysis also suggests that, even after the end of free national testing in England, the reduction in global synchrony with CIS estimates was minimal and did not undermine the reliability of overall trends. These findings support the validity of the Virus Watch approach as a reliable source of proxy estimates of community-level incidence and prevalence. Furthermore, the Flu Watch study, on which our study methodology was based, demonstrated the effectiveness of this approach for monitoring influenza transmission during and after the H1N1 pandemic in England without extensive testing systems. Flu Watch–estimated PCR-confirmed influenza rates were, on average, 22 times higher (95% CI 17‐28) than rates reported through primary-care–based surveillance during the winter seasons from 2009 to 2011 [9]. The adaptable and cost-efficient design of Virus Watch ensures its sustainability beyond peak outbreaks, enabling the monitoring and guiding of target responses to emerging variants and future acute respiratory diseases. While our findings suggest that infection trends can be robustly monitored even in the absence of a national testing program, further investigation is needed to determine whether the Virus Watch approach can be effectively adapted for use in low-resource settings.

### Limitations

Several limitations should be considered. First, due to differences in study design, we were unable to directly compare COVID-19 positivity and incidence rates with ONS CIS, as we could not use the same methodology for estimating these rates. This is because we were unable to model the clearance time, as ONS CIS did, since participants do not test regularly for COVID-19. As a result, we lack data on the date they stop testing positive. While the difference in methodology limits the comparability of our estimates, the similarity observed between the 2 studies remains encouraging. In addition, Virus Watch likely underestimated positivity and incidence rates, as it mainly captured symptomatic cases and did not incentivize swab returns or weekly reporting, in contrast to ONS CIS. The accuracy of our estimated rates may also be impacted by the self-reporting nature of our study, since reports serve as the first recorded sign of infection, which may not align with the actual onset of infection. Furthermore, the smaller sample size in our study also affects the generalizability of our findings, particularly in the lower age groups.

The self-selected nature of the Virus Watch cohort, while broadly representative of the general English population, introduces potential biases. Our cohort is likely biased toward individuals with a greater interest in COVID-19 and health research, potentially skewing toward a more cautious and health-conscious demographic. In addition, the underrepresentation of younger and the oldest age groups and ethnic minority groups, particularly Black and other Asian groups, and the exclusion of households with more than 6 members and individuals residing in institutional settings further restricts the generalizability of our COVID-19 estimates. Furthermore, the significant attrition observed over the 3-year study period, particularly among younger, ethnically diverse, and London-based participants, underscores the challenges of long-term cohort studies [8]. While this pattern is consistent with other longitudinal studies, this limitation may impact the representativeness of the cohort over time at faster rates compared to ONS CIS due to their continual incentivization [32].

### Conclusion

In this study, we used Virus Watch data to estimate COVID-19 positivity, incidence, and hospitalization rates in England and Wales, comparing our findings with estimates from ONS CIS and SARI. Despite differences in study design, Virus Watch demonstrated strong correlations with ONS CIS infection rates, highlighting its usability as a complementary surveillance method. Each surveillance system has unique strengths and limitations, and our findings underscore the need for complementary surveillance approaches to address gaps in individual systems. Virus Watch’s sustainable and low-cost design is suited for settings without extensive national testing infrastructure. Beyond COVID-19, this surveillance approach may also effectively track other acute respiratory infections, guiding public health responses in both high- and low-resource settings.

## Supplementary material

10.2196/69655 Multimedia Appendix 1 Supplementary figures showing the 9-week rolling Spearman ⍴ correlation coefficients over time of COVID-19 positivity, incidence, and hospitalization rates in England and Wales.

## Abbreviations

CIS: Community Infection Survey
HES: Hospital Episode Statistics
LFT: lateral flow test
NHS: National Health Service
ONS: Office for National Statistics
PCR: polymerase chain reaction
REACT: Real-Time Assessment of Community Transmission
SARI: Severe Acute Respiratory Infections Watch
SGSS: Second Generation Surveillance System
