# Correction: Comparison of Virus Watch COVID-19 Positivity, Incidence, and Hospitalization Rates With Other Surveillance Systems: Surveillance Study

**DOI:** 10.2196/85512

**Published:** 2025-10-10

**Authors:** Wing Lam Erica Fong, Vincent Grigori Nguyen, Sarah Beale, Thomas E Byrne, Cyril Geismar, Ellen Fragaszy, Jana Kovar, Alexei Yavlinsky, Ibrahim Abubakar, Andrew C Hayward, Robert W Aldridge

**Affiliations:** 1 Institute of Health Informatics, University College London, 222 Euston Road, London, NW1 2DA, United Kingdom, 44 02035495969; 2 Institute of Epidemiology and Health Care, University College London, London, United Kingdom; 3 Department of Population, Policy and Practice, UCL Great Ormond Street Institute of Child Health, London, United Kingdom; 4 NIHR Health Protection Research Unit in Modelling and Health Economics, Department of Infectious Disease Epidemiology, School of Public Health, Imperial College London, London, United Kingdom; 5 Department of Infectious Disease Epidemiology, London School of Hygiene & Tropical Medicine, London, United Kingdom; 6 Faculty of Population Health Sciences, University College London, London, United Kingdom; 7 NIHR Health Protection Research Unit in Health Analytics and Modelling, Department of Infectious Disease Epidemiology, School of Public Health, Imperial College London, London, United Kingdom; 8 Institute for Health Metrics and Evaluation, University of Washington, Seattle, United States

In “Comparison of Virus Watch COVID-19 Positivity, Incidence, and Hospitalization Rates With Other Surveillance Systems: Surveillance Study” [1], the authors noted one error.

Coauthor ACH was mistakenly linked to the fourth affiliation, which has now been removed from the author. The following affiliation was added:

NIHR Health Protection Research Unit in Health Analytics and Modelling, Department of Infectious Disease Epidemiology, School of Public Health, Imperial College London, London, United Kingdom

This will appear as the seventh affiliation and be attached to ACH, with any subsequent affiliations being renumbered accordingly.

The correction will appear in the online version of the paper on the JMIR Publications website, together with the publication of this correction notice. Because this was made after submission to PubMed, PubMed Central, and other full-text repositories, the corrected article has also been resubmitted to those repositories.
