# Supplementary material for: Comparison of Virus Watch COVID-19 Positivity, Incidence, and Hospitalization Rates With Other Surveillance Systems: Surveillance Study
Source: JMIR Public Health Surveill. 2025 Sep 29;11:e69655. doi: 10.2196/69655 (PMC12479050; doi:10.2196/69655)

**Supplementary Figure 1. 9-week rolling Spearman’s rho correlation coefficients over time of COVID-19 positivity and incidence rates in England and Wales.** The red line indicates the end of free national COVID-19 testing.

|  | ONS CIS Positivity Rates | ONS CIS Incidence Rates |
| --- | --- | --- |
| England (with linked data) | 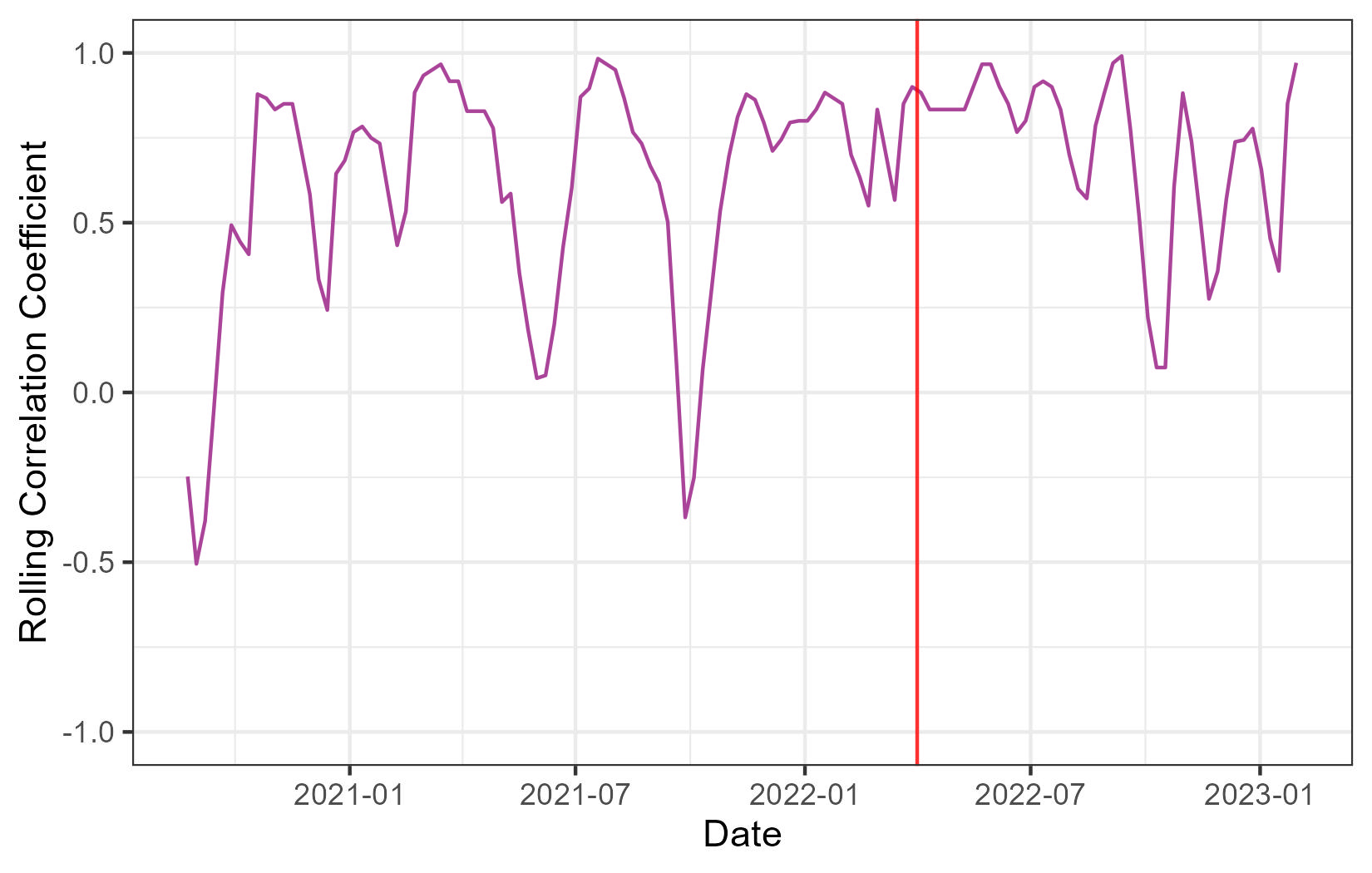 | 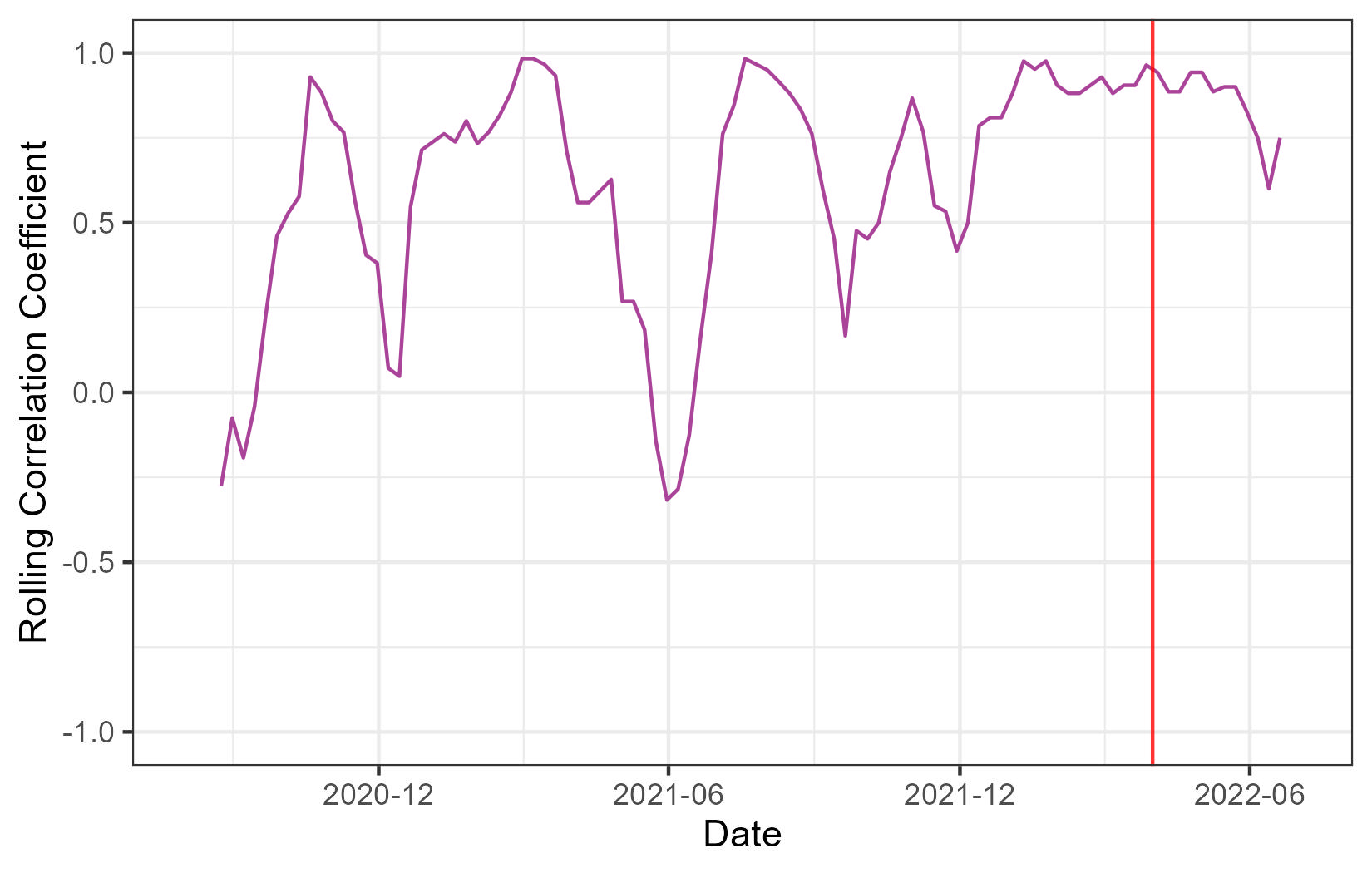 |
| England (no linked data) | 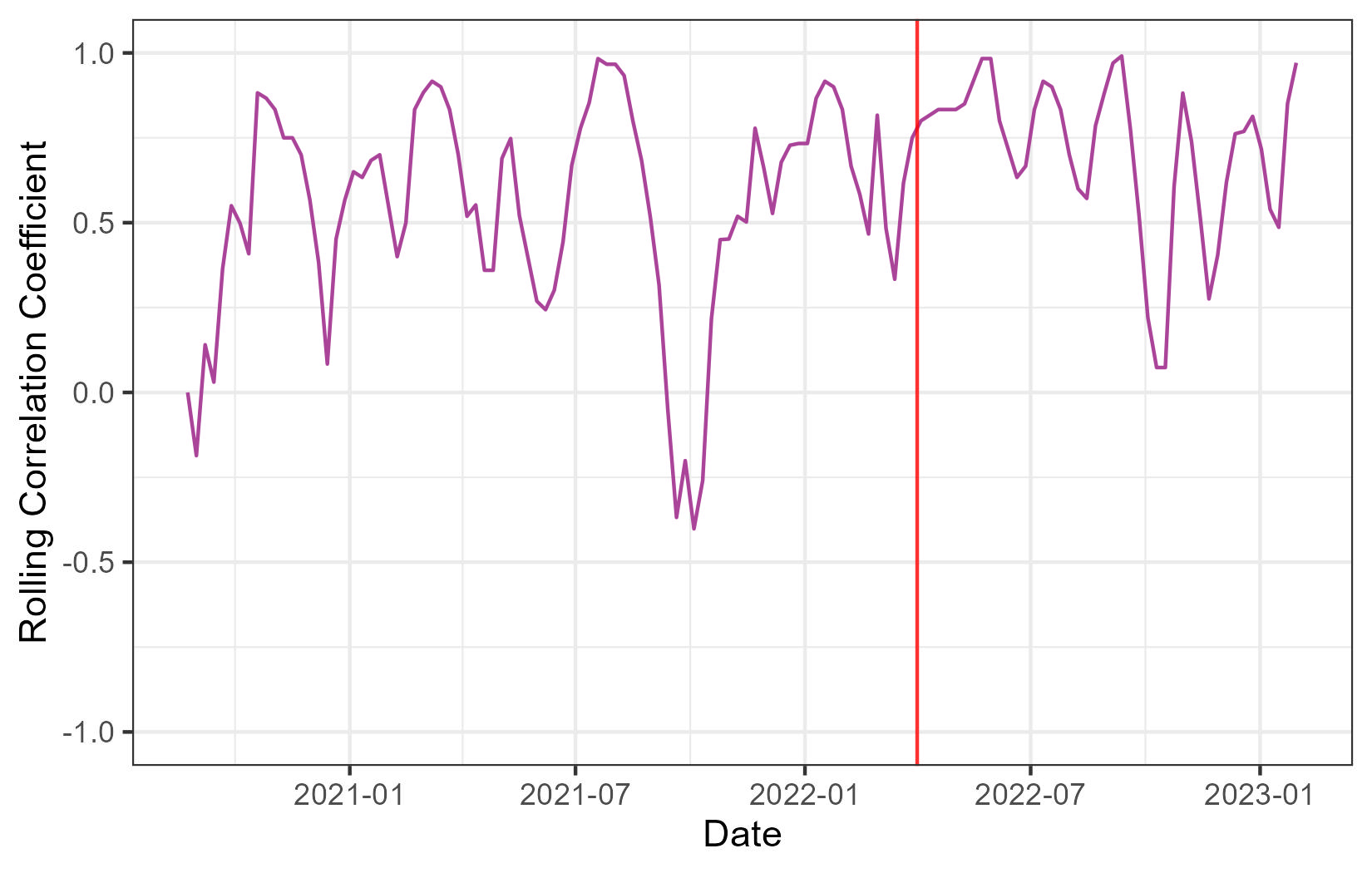 | 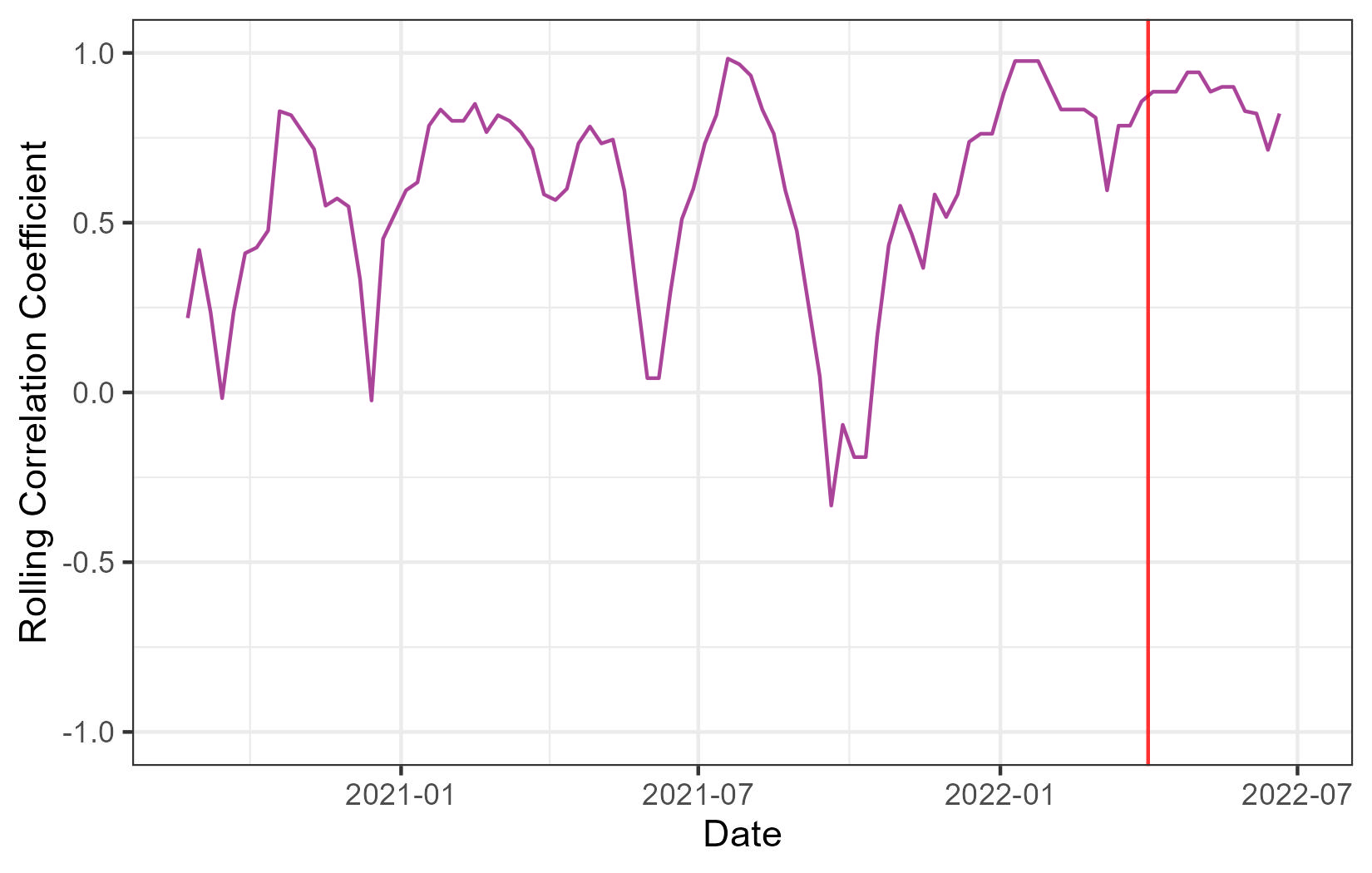 |
| Wales | 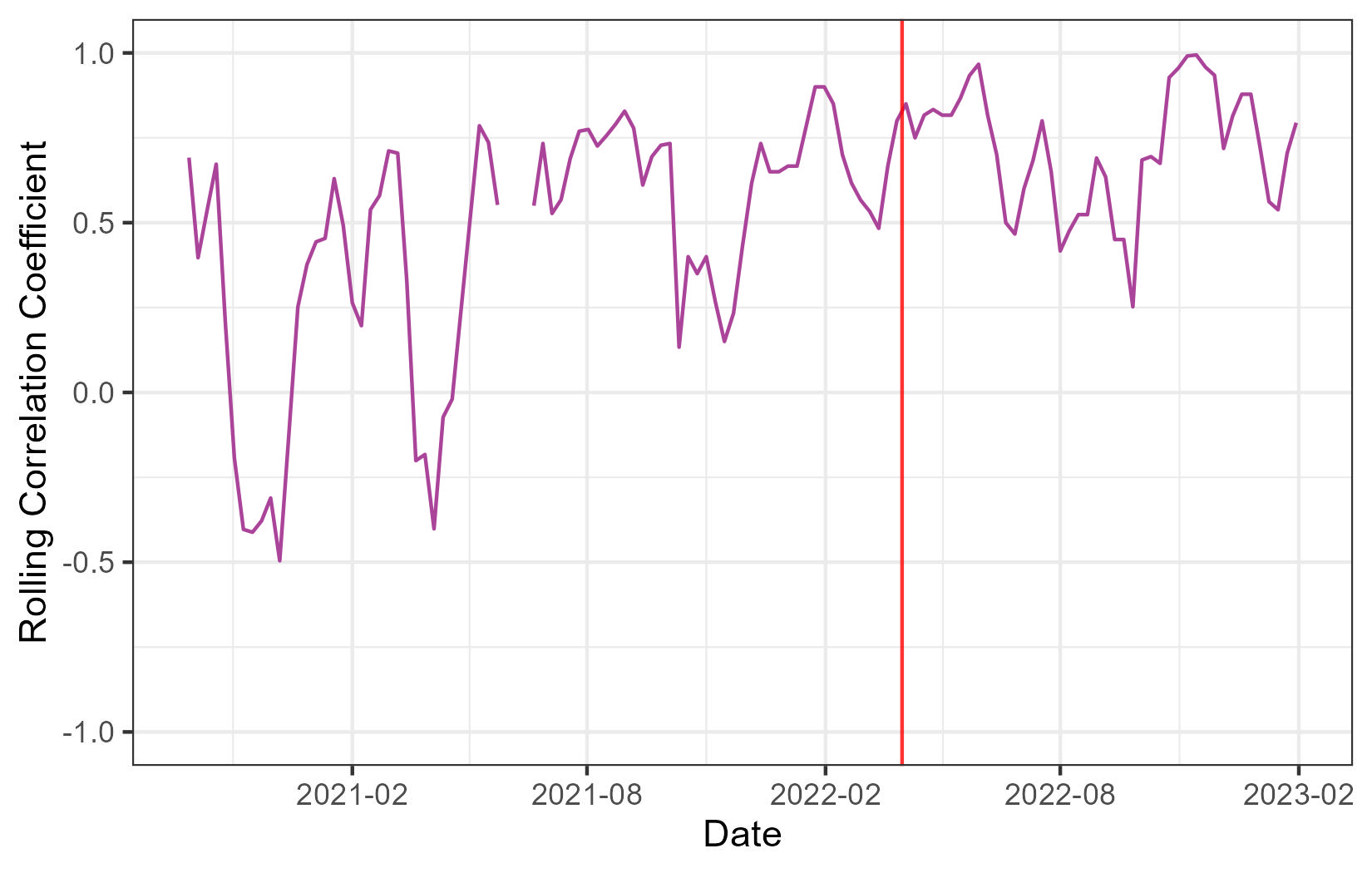 | 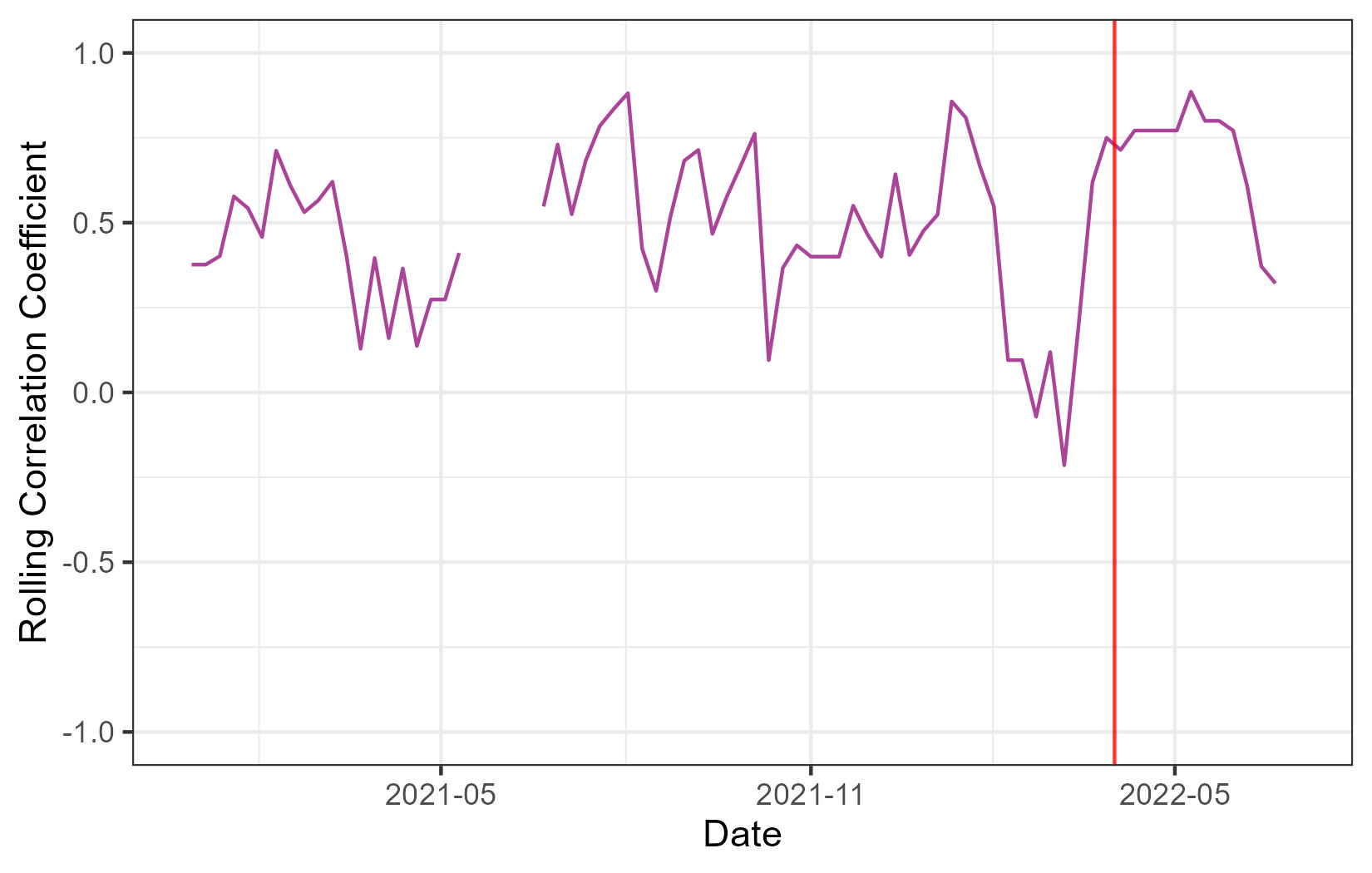 |

**Supplementary Figure 2. 9-week rolling Spearman’s rho correlation coefficients over time of COVID-19 hospitalisation rates in England.**


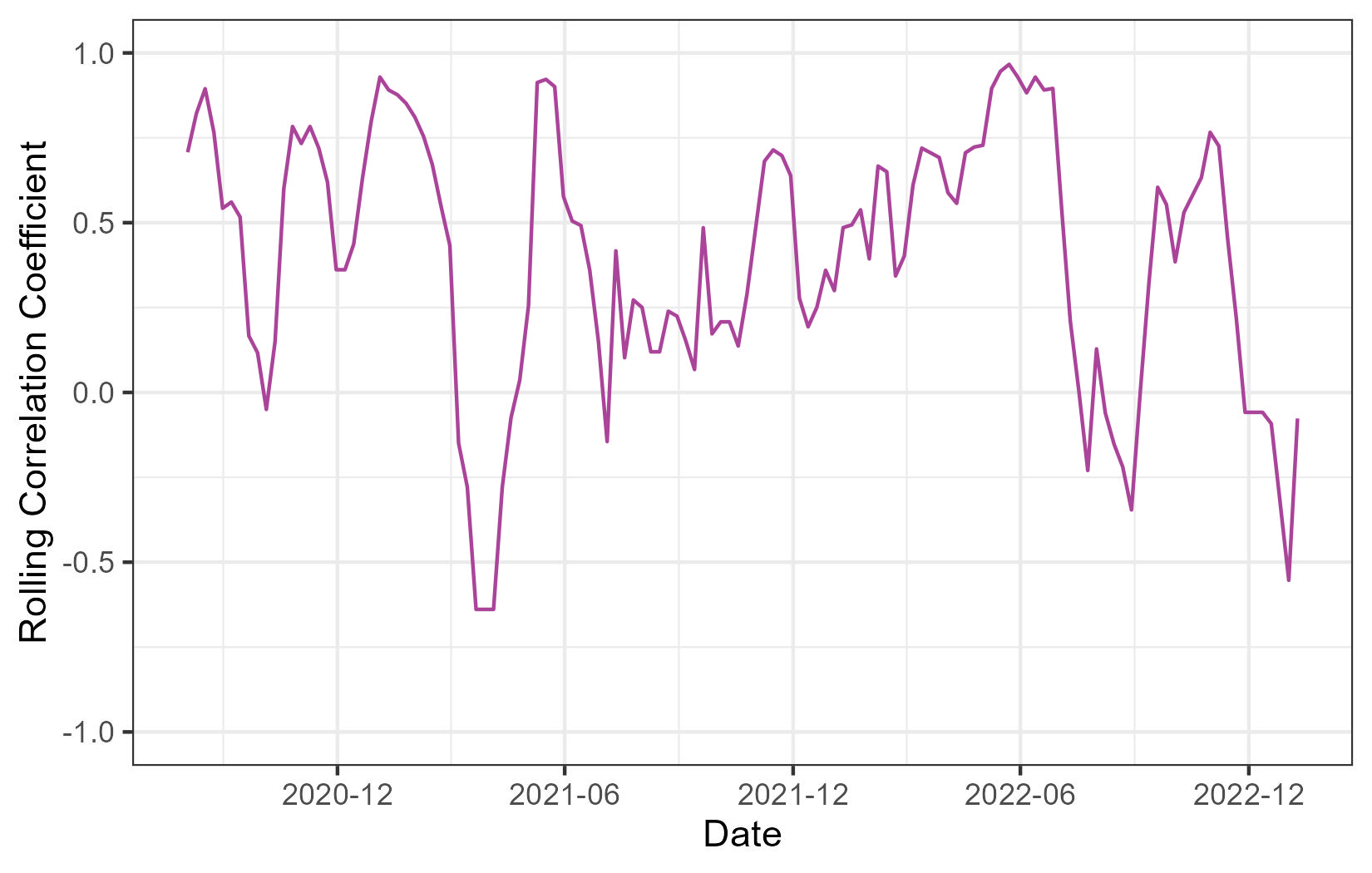

Supplement: Multimedia Appendix 1 [file publichealth-v11-e69655-s001.docx]
